# Radioiodine Therapy of Graves’ Disease in Women with Childbearing Potential and the Pre-Conceptional Counseling About Antithyroid Drugs

**DOI:** 10.3390/jcm14051667

**Published:** 2025-02-28

**Authors:** Markus Dietlein, Matthias Schmidt, Alexander Drzezga, Carsten Kobe

**Affiliations:** Department for Nuclear Medicine, University Hospital Cologne, Kerpener Str. 62, 50937 Cologne, Germany

**Keywords:** radioiodine therapy, Graves’ disease, TSH receptor antibodies, fetal hyperthyroidism, propylthiouracil, thiamazole

## Abstract

Graves’ disease and hyperthyroidism in women with childbearing potential are a challenge in pre-conceptional counseling. The non-surgical alternatives are radioiodine therapy or antithyroid drugs. Here, we focus on the TSH receptor antibody (TRAb) level—without or after radioiodine therapy—and the probability of fetal or neonatal hyperthyroidism. This immunological effect should be weighed against the risk of congenital malformation taking propylthiouracil during pregnancy. For up to 2 years after radioiodine therapy for Graves’ disease, TRAb levels may remain above the pre-therapeutic level. The time of conception after radioiodine therapy and a high TRAb level are associated with the likelihood of neonatal hyperthyroidism: 8.8% probability if conception occurred 6–12 months after radioiodine therapy, with a 5.5% probability for 12–18 months, and 3.6% probability for 18–24 months. The TRAb value above 10 U/L in the third trimester is the main risk factor for neonatal hyperthyroidism. If a woman does not wish to postpone her family planning, the pre-conceptional counseling has to describe the risk of propylthiouracil, thiamazole, or of an uncontrolled hyperthyroidism. According to some national cohort studies (Danish, Swedish, Korean), the risk for fetal malformations (ear, urinary tract) under propylthiouracil is increased by 1.1–1.6%, in addition to the spontaneous risk for unexposed pregnant women. For thiamazole, the additional risk for fetal malformation was about 2–3%, depending on the dose of thiamazole. Propylthiouracil has posed a lower risk for congenital malformation than an uncontrolled hyperthyroidism. To minimize the risk for the newborn, women with Graves’ disease and hyperthyroidism should offer a definitive therapy strategy (e.g., radioiodine therapy) long before planning a pregnancy.

## 1. Introduction 

Since the 1940s, radioiodine (I-131) has been used to treat thyroid diseases. The concept of ablative radioiodine therapy for Graves’ disease with hyperthyroidism (higher intended dose) and of function-optimized radioiodine therapy for toxic or non-toxic goiter (lower intended dose to the thyroid, dosimetric approach pre-therapeutically) and has remained unchanged over a long period [1]. Several subpopulations with benign thyroid diseases and specific circumstances are candidates for radioiodine therapy (Table 1) [2,3]:

- Patients with Graves’ disease (GD) are sent to radioiodine therapy when the first-line therapy has failed. The first manifestation of hyperthyroidism in Graves’ disease is treated usually with carbimazole or thiamazole for 12–24 months. The individual timing to stop antithyroid medication depends on the TSH receptor antibody level (TRAb preferably <2 U/L before attempting withdrawal), the dosage of the antithyroid drug (preferably ≤2.5 mg carbimazole or thiamazole), and the thyroid function setting (TSH preferably ≥0.3 mU/L) [4]. Radioiodine therapy in Graves’ disease with small goiter is the preferred option in recurrent hyperthyroidism within 2 years after withdrawal of antithyroid drugs.
- An indication for an early radioiodine therapy in Graves’ disease is the persistent TRAb value above 10 U/L after six months of antithyroid therapy. In this scenario, the success probability of conservative therapy is below 5% [5]. Radioiodine therapy can be offered first line when the risk of recurrent hyperthyroidism is high. The main risk factors are the young age at initial presentation with Graves’ disease, the large goiter of >40 mL, and smoking.
- In subclinical hyperthyroidism (TSH < 0.1 mU/L), radioiodine therapy in Graves’ disease is recommended in patients >65 years old or in patients with atrial fibrillation/cardiovascular comorbidity. An individual indication for radioiodine therapy in older patients is the TSH range of 0.1–0.4 mU/L [4].
- Radioiodine therapy can be administered safely in adolescents, preferably those over 15 years [4,6,7]. The duration of the antithyroid medication with carbimazole or thiamazole should be longer in adolescents than in adults and can be extended to at least 3 years (up to 6 years) in adolescents [6]. Due to the increased risk of liver failure, propylthiouracil is contraindicated in adolescents.
- In special circumstances, radioiodine therapy is an option for thyroid remnant ablation if postoperative residual thyroid tissue remains and there is an advanced florid endocrine orbitopathy (“Moleti concept”). As endogenous TSH stimulation by temporarily discontinuing thyroid medication is not considered, exogenous TSH stimulation by rhTSH is performed as “off-label use” in the usual dosage of rhTSH, followed by the administration of a fixed radioiodine activity of 1.1 GBq ^131^I [8].

The immunological effects of radioiodine therapy in younger women with Graves’ disease regarding family planning, pre-conceptional counseling, possible pregnancy complications (e.g., fetal or neonatal hyperthyroidism), and the teratogenic side effects of antithyroid drugs are discussed in this review [9,10,11]. We present the literature on TRAb after radioiodine therapy, on TRAb during pregnancy, and on antithyroid drugs during pregnancy with a search in the database of PubMed^®^.

## 2. Graves’ Disease—Immunological Aspects in Women with Childbearing Potential

How can be Graves’ disease in women of childbearing potential managed (Table 2)? For up to 2 years after radioiodine therapy for Graves’ disease, TSH receptor antibody levels (TRAb) may remain above the pre-therapeutic level [9]. TSH receptor antibodies are going transplacentar and could lead to fetal/neonatal hyperthyroidism if the TRAb levels exceed at least 5 U/L or are increased at least by a factor of ≥3 above the upper limit in the case of stimulating TRAb in the last trimester [12,13,14]. However, the frequently cited TRAb level above 5 U/L is a very conservative assumption.

The average risk of fetal hyperthyroidism is about 1–2% of mothers with Graves’ disease, manifesting beyond the 22–26 week’ gestation [15]. The risk of fetal hyperthyroidism is much higher (approximately 5–20%) when the pregnancy starts with maternal TRAb levels of more than 30–40 U/L. Signs of fetal hyperthyroidism include tachycardia (>160 beats per min), goiter, oligohydramnion, growth restriction, premature bone ossification, and fetal hydrops. Fetal ultrasound surveillance is recommended in mothers with elevated TRAb above 5 U/L or with uncontrolled hyperthyroidism.

A literature review by van Dijk et al. [13] covering six cohort studies examined the individual TRAb levels of pregnant women diagnosed with fetal or neonatal hyperthyroidism. TRAb levels were measured using first to third-generation assays, so the authors related maternal TRAb levels to the upper reference limits of each assay used. As only one of the six cohort studies had consecutive patient inclusion, a selection bias limits the evidence. In the result, 31 cases of fetal/neonatal hyperthyroidism were described, with maternal TRAb levels in the six cited cohort studies being elevated by a factor of 3.7, 4, 9.2, 19.7, 26.7, and 32, respectively, above the upper limit. Inconsistent measurement times during the trimesters also limit the definition of a pre-conceptional TRAb levels requiring monitoring. 

As a part of the above-mentioned review, the monocentric French study by Abeillon-du Payrat et al. is frequently cited: 47 newborns were described whose mothers had elevated TRAb levels (second-generation assay) during pregnancy [12]. Seven of these women had previously undergone radioiodine therapy. Nine of the 47 newborns had laboratory-confirmed hyperthyroidism at birth, including five newborns requiring treatment (antithyroid medication for 14–80 days, median 60 days). No neonatal hyperthyroidism was observed below a maternal TRAb level of 5.6 U/L (measured in the second trimester). The likelihood of neonatal hyperthyroidism increased with high TRAb levels. Laboratory evidence of neonatal hyperthyroidism was associated with relatively high maternal TRAb levels, with a median of 26.9 U/L (mean 62.4 U/L). Among the 35 euthyroid newborns, maternal TRAb levels ranged from 1 to >40 U/L, median 5.2 U/L, mean 8.2 U/L. Three newborns were hypothyroid, indicating blocking TSH receptor antibodies. The French authors concluded that close fetal monitoring is warranted if maternal TRAb levels exceed 5 U/L (measured in the study in the second trimester). 

A monocentric Japanese study by Yoshihara et al. [14] analyzed the likelihood of neonatal hyperthyroidism if pregnancy occurred within 2 years after radioiodine therapy for Graves’ disease. Despite the radioiodine therapy, 54 of the 145 pregnant women (37%) required either antithyroid medication due to persistent hyperthyroidism or high-dose iodine as another treatment concept for Graves’ disease with overt hyperthyroidism in Japan. Overall, 8 of the 145 newborns showed laboratory-confirmed neonatal hyperthyroidism, corresponding to a probability of 5.5%. Four of the eight hyperthyroid newborns required antithyroid medication. Seven of the eight neonatal hyperthyroidism cases were associated with an unsuccessful ablation after radioiodine therapy in the mother before family planning. The following association was found between the time of conception after radioiodine therapy and the likelihood of neonatal hyperthyroidism: 8.8% probability if conception occurred 6–12 months after radioiodine therapy, 5.5% probability for 12–18 months, and 3.6% probability for 18–24 months (Table 3). Multivariate analysis identified only the TRAb value in the third trimester as risk factor for neonatal hyperthyroidism. In the ROC analysis, a TRAb threshold of 9.7 IU/L for the third trimester was calculated. Below a TRAb value of 9.7 IU/L (third trimester), no neonatal hyperthyroidism was observed. It is important to note that TRAb levels typically decrease during the whole duration of pregnancy. In the first trimester, maternal TRAb levels among the eight women whose newborns later had neonatal hyperthyroidism ranged from 30.6 IU/L to 40 IU/L, with seven values documented at 40 IU/L. The study also reported pre-therapeutic TRAb levels: In the 137 patients who later gave birth to euthyroid newborns, the median pre-therapeutic TRAb level was 9.95 IU/L (range 0.3–40 IU/L), while in the eight patients whose newborns later had neonatal hyperthyroidism, the median TRAb level was 40 IU/L (range 10.2–40 IU/L). Retrospective data suggest that a high TRAb level at initial diagnosis, an unsuccessful ablation after radioiodine therapy, and a short interval between radioiodine therapy and conception increase the likelihood of neonatal hyperthyroidism. 

In the pre-conception counseling, we should inform patients of an increase in transplacental TSH receptor antibodies within 2 years after radioiodine therapy. If TRAb levels exceed 5–6 U/L or are >3 times the upper reference limit during pregnancy, fetal monitoring (fetal sonography, fetal pulse monitoring) is advised. For newborns after a pregnancy with high maternal TRAb levels, the pediatric and endocrinological literature recommends a TRAb measurement from umbilical cord blood for risk stratification [16,17].

Ideally, women with Graves’ disease and hyperthyroidism planning pregnancy would receive definitive therapy long before pregnancy [18]. If possible, radioiodine therapy should be performed early enough to delay family planning for 2 years, at least for 6 months. The treatment aim after radioiodine therapy is stable euthyroidism. There is no specific treatment to achieve normalization of TRAb pre-conception [15]. If time is pressing to get pregnant, the guidelines does not recommend a specific therapy, as alternatives like thyroidectomy or antithyroid medication with propylthiouracil also carry specific treatment risks.

## 3. Can Propylthiouracil Be an Alternative to Radioiodine Therapy?

A warning about the teratogenic potential has been formulated for thiamazole and carbimazole (Table 4). The teratogenic risk appears to correlate with the dosage of carbimazole or thiamazole in the first trimester; the maternal hyperthyroidism by itself is not associated with these anomalies [19]. What is the data for propylthiouracil? According to the Danish cohort study, a risk for fetal malformations of 8.3% was observed under propylthiouracil (*n* = 889 pregnant women) compared to a spontaneous risk for unexposed pregnant women (*n* = 1,159,181 pregnant women) of 6.7%. The hazard ratio for propylthiouracil was calculated at 1.17 with a 95% confidence interval of 0.91–1.49, indicating that the statistical significance for an increased risk for fetal malformations was not reached for propylthiouracil [10]. For thiamazole (*n* = 1574 pregnant women), the risk for fetal malformation was calculated at 9.6%, with a hazard ratio of 1.41 and a 95% confidence interval of 1.19–1.67, indicating a statistically significant risk for thiamazole compared to the spontaneous risk for fetal malformation in thyroid-healthy women without medication. In the Swedish cohort study, the spontaneous risk for birth defects was 8.04%, and the calculated risk for birth defects for pregnant women on propylthiouracil was 6.42% only [11]. However, in the Swedish cohort study, statistically increased malformations of the ear (ICD-10: Q17) and urinary tract (ICD-10: Q62) were observed in pregnant women on propylthiouracil. More evidence is needed to establish the degree of risk.

A Korean cohort study confirmed the estimated risk for thiamazole und propylthiouracil, based on 2,886,970 newborns, from whom 12,891 newborns were exposed to antithyroid drugs [20]. The spontaneous prevalence of congenital malformations was 5.94% without antithyroid drugs in the control group, 7.04% with propylthiouracil, 8.13% with thiamazole, and 7.98 with propylthiouracil and thiamazole. The risk difference per 1.000 birth was calculated as 8.81 under propylthiouracil, 17.05 under thiamazole, and 16.53 under propylthiouracil and thiamazole. Switching from propylthiouracil to thiamzole increased the risk of malformation significantly by the odds ratio 1.79 (Table 5). The duration and the cumulative dose of propylthiouracil were not associated with the observed risk of malformations. Prescribing thiamazol, the cumulative dose of 495 mg during the first trimester increased the risk of congenital malformations by the odds ratio 1.87 [20].

## 4. Has the Uncontrolled Hyperthyroidism a Teratogen Potential?

Larger case control studies from newborns with congenital malformations (e.g., choanal atresia) whose mothers had either uncontrolled hyperthyroidism without taking medication or whose mothers had taken antithyroid drugs indicated that hyperthyroidism is a separate risk factor [21,22]. The risk of malformations was statistically even higher for the group with an untreated hyperthyreoidism than for the group with a controlled thyroid function under methimazol [22]. The risk of choanal atresia affects the gestational weeks 9 and 10 [21]. The meta-analysis by Agrawal et al. confirmed the risk of an untreated hyperthyroidism with a risk factor of 1.04 [23]. Compared to an untreated hyperthyroidism, propylthiouracil decreased the risk of congenital malformations by the factor 0.69, whereas the risk of thiamazole did not differ significantly from the risk of an uncontrolled hyperthyroidism. The evidence of the studies is weakened by patient heterogeneity. A possible bias is the risk of birth defects in children whose mothers have taken an inappropriately high dose of antithyroid drugs with the consequence of overt hypothyroidism.

## 5. Timing of Radioiodine Therapy

Since radioiodine therapy is usually an elective treatment, optimal conditions should be ensured when scheduling to minimize radiation exposure. To minimize radiation exposure to the breasts, the recommended interval between the end of breastfeeding and the start of radioiodine therapy has been extended to 3 months, in line with the recommendation for radioiodine diagnostics in differentiated thyroid carcinoma [24]. The recommended interval between the administration of iodine-containing contrast media and the start of radioiodine therapy is (at least) 2 months. For the application of specific medications (amiodarone), a much longer period may be required before a successful radioiodine therapy can be performed.

## 6. Discussion

The association between antithyroid drugs and births defects bases on nationwide studies with larger sample sizes of exposed women (e.g., >500 women) [23]. For women on thiamazole who are planning conception soon and who require antithyroid drugs, we recommend changing to propylthiouracil pre-conception [15]. Antithyroid drug treatment carries a small risk of teratogenic side-effect in early pregnancy, especially in weeks 5–11 [18]. The recent meta-analysis by Agrawal et al. has described a high number of anomalies for exposure to both thiamazole and propylthiouracil and does not support the strategy to switch from thiamazole to propylthiouracil in the first trimester [23]. Propylthiouracil is potentially hepatotoxic, with a small risk of fulminant liver failure. Therefore, propylthiouracil is recommended as first choice only in the planning of and during pregnancy. Discontinuation of treatment with propylthiouracil may be feasible at the end of the second trimester or in the third trimester due to the disappearance of maternal TRAb [19]. Very low doses of propylthiouracil (e.g., 1/4 tablet in the morning and 1/4 tablet in the evening) have the advantage that the risk of recurrence will be minimized.

Another consideration is the risk of birth defect by hypothyroidism of the mother or the fetus. To prevent fetal hypothyroidism, the dosage of propylthiouracil should not exceed 150 mg in the third trimester [25]. If switched to thiamazole after the first trimester (despite the data of the Korean cohort study), the dosage should not exceed 10 mg thiamazole in the third trimester. These dosage recommendations are focused on the aspect of fetal hypothyroidism and were statistically determined using ROC curves, but do not cover the risk of embryopathy. A fetal hypothyroidism rarely causes fetal goiter with tracheal and esophageal compression, polyhydramnion, and complication at delivery. Therefore, the dosage of the antithyroid drugs should be as low as possible, and maternal fT4 is maintained in the high–normal range [15]. In women with mild disease, the termination of antithyroid drugs should be considered in each trimester.

What are the consequences, when a young woman with Graves’ disease will ask for non-surgical treatment options under the aspect of family planning? A TRAb-induced fetal or neonatal hyperthyroidism is better to treat than a congenital malformation induced by antithyroid drugs. The probability of fetal hyperthyroidism, manifesting beyond the 22–26 week’ gestation, is high (approximately 5–20%) when the pregnancy starts with maternal TRAb levels of more than 30–40 U/L. Women with childbearing potential and such high TRAb levels are not suitable candidates for radioiodine therapy. The maternal TRAb levels typically decrease during the whole duration of pregnancy. The TRAb value in the third trimester is crucial for the probability of neonatal hyperthyroidism. A close fetal monitoring is warranted if maternal TRAb levels exceed 10 U/L, measured in the third trimester. The maternal TRAb will remain in the newborns for several months, whereas the maternal antithyroid drugs are cleared from the newborns rapidly after delivery [15]. If maternal TRAb are elevated in the third trimester, neonatal TSH and fT4 should be measured at delivery, then on days 3–5, and on days 10–14 postpartum or when the newborn develops symptoms. Neonatal hyperthyroidism can be easily missed if relying on newborn screening of TSH alone, as TSH screening is optimized to screen for hypothyroidism. For a newborn with symptomatic hyperthyroidism, thiamazole is allowed, propylthiouracil has a hepatotoxic potential.

## 7. Conclusions

Patients with Graves’ disease, small goiter, and moderately measurable TRAb levels are suitable candidates for radioiodine therapy. After a waiting period of 6 months, the risk of increased TRAb after radioiodine therapy (fetal hyperthyroidism) is no higher than the risk of propylthiouracil (congenital malformation). Prospective data from a head-to-head comparison are missing.

## Figures and Tables

**Table 1:** Main features of the three available treatment strategies for hyperthyroidism due to Graves’ disease or toxic goiter.

	Antithyroid Drugs	Surgery	Radioiodine
Time to initial improvement	2–4 weeks	Needs antithyroid drug pre-treatment (toxic goiter) Success immediately	Needs antithyroid drug pre-treatment; 4–8 weeks after RIT
Recurrence	90–100% (toxic goiter)	1–4%	5–10%
Use in pregnancy/Breast feeding	Yes (First trimester: PTU)	Second trimester	No
Adverse effect on eye disease in toxic goiter	No	No	0.1% (1% risk of immune hyperthyroidism; rarely eye symptoms)
Adverse effect on eye disease in Graves’ disease	Spontaneous course	Spontaneous course	Glucocorticoids in patients with Graves’ ophthalomopathy
Interference with daily activities	No	Requires hospital admission	Need for radiation precautions and in many countries requires hospital admission
Key adverse effects	Rash; arthralgia; hepatitis; agranulocytosis	Surgical; vocal cord paralysis; hypoparathyroidism; hypothyroidism	Hypothyroidism (>95% with an ablative dose concept, 10% with a low-dose concept)

**Table 2:** Main features of the three available treatment options for Graves’ disease with hyperthyroidism in women with childbearing potential. Abbreviations: ATD, antithyroid drugs; PTU, propylthiouracil; RIT, radioiodine therapy; TRAb, TSH receptor antibody.

	Antithyroid Drugs	Surgery	Radioiodine Therapy
Efficacy	Euthyroid function within 1–2 months. Thiamazole or carbimazole as standard. PTU pre-conceptional and during the first trimester of pregnancy	Euthyroid function with levothyroxine; Increase in dosage during the first and second trimester of pregnancy	Euthyroid function with levothyroxine achieved about 3–4 months after RIT
Key adverse effects in the pre-conceptional state or in pregnancy	Hepatotoxicity of PTU. Birth defects in early pregnancy with thiamazole 2–3% higher than the spontaneous risk, with PTU 1.1–1.6%	Surgical complications: vocal cord paralysis; hypoparathyroidism	TRAb elevation for up to 2 years after RIT. Fetal or neonatal hyperthyroidism, if TRAb > 10 U/L in the third trimester
Practical considerations	Pre-conceptional conseling. Change from thiamazole to PTU during the first trimester bears risk of birth defects. Low dose of PTU. Doses > 150 mg PTU induce fetal hypothyroidism	Feasible in second trimester, if refractory to ATD or adverse effects of ATD	feasible only before family planning; High TRAb levels in the third trimester require fetal monitoring

**Table 3:** Data from Japan on the incidence of neonatal hyperthyroidism when mothers with Graves’ disease were treated with radioiodine. In the group with neonatal hyperthyroidism the mothers had a median thyroid volume of 62 mL and a median TRAb of 40 U/L when radioiodine therapy was started [14]. This is generally a negative selection with an increased probability that the first radioiodine therapy cannot eliminate hyperthyroidism. Abbreviation: TRAb, TSH receptor antibody.

Conception After Radioiodine Therapy	Incidence of Neonatal Hyperthyroidism	TRAb Values at Radioiodine Therapy in Mothers of Neonates with Hyperthyroidism
Within 6–12 months	3/34 (8.8%)	40 U/L, 40 U/L, 16.2 U/L
Within 12–18 months	3/55 (5.5%)	40 U/L, 40 U/L, 11.5 U/L
Within 18–24 months	2/56 (3.6%)	40 U/L, 10.2 U/L

**Table 4:** Nationwide cohort studies on the incidence of births defects in unexposed women and after use of antithyroid drugs in early pregnancy [10,11,20]. In the Korean cohort study, the risk of congenital malformations by methimazole was dose dependent.

Cohort	Unexposed Women	Methimazole, Thiamazole Alone	Propylthiouracil Alone
Denmark 1997–2016	77,791/1,159,181 (6.7%)	151/1574 (9.6%)	74/889 (8.3%)
Sweden 2005–2014	54,827/682,343 (8.04%)	11/162 (6.79%)	14/218 (6.42%)
South Korea 2008–2014	170,716/2,872,109 (5.94%)	91/1120 (8.13%)	699/9930 (7.0%)

**Table 5:** Data from the Korean cohort study on the incidence of congenital malformations when the antithyroid drugs were switched in the period between pre-pregnancy and the first trimester [20]. Data on more accurate exposure timing will be necessary to verify the risk associated with switching antithyroid drugs. Abbreviations: MMI, methimazole; PTU, propylthiouracil.

Switch	Switch Group	Comparative Value
from methimazole to propylthiouracil	166/2079 (7.98%)	MMI continued: 70/909 (7.70%)
from propylthiouracil to methimazole	18/158 (11.39%)	PTU continued: 357/5184 (6.89%)

## References

[1] Dietlein M., Grünwald F., Schmidt M., Kreissl M.C., Luster M. (2024). Guideline for radioiodine therapy for benign thyroid diseases (6/2022-AWMF No. 031-003). German. Nukl..

[2] Weetman A.P. (2007). Radioiodine treatment for benign thyroid diseases. Clin. Endocrinol..

[3] Campenni A., Avram A.M., Verburg F.A., Iakovou I., Hänscheid H., de Keizer B., Ovčariček P.P., Giovanella L. (2023). The EANM guideline on radioiodine therapy of benign thyroid disease. Eur. J. Nucl. Med. Mol. Imaging.

[4] Kahaly G.J., Bartalena L., Hegedüs L., Leenhardt L., Poppe K., Pearce S.H. (2018). 2018 European Thyroid Association guideline for the management of Graves’ hyperthyroidism. Eur. Thyroid J..

[5] Eckstein A., Mann K., Kahaly G.J., Grußendorf M., Reiners C., Feldkamp J., Quadbeck B., Bockisch A., Schott M. (2009). Role of TSH receptor autoantibodies for the diagnosis of Graves’ disease and for the prediction of the course of hyperthyroidism and ophthalmopathy. Recommendations of the Thyroid Section of the German Society of Endocrinology. Med. Klin..

[6] Léger J., Carel J.C. (2017). Management of endocrine disease: Arguments for the prolonged use of antithyroid drugs in children with Graves’ disease. Eur. J. Endocrinol..

[7] Ross D.S., Burch H.B., Cooper D.S., Greenlee M.C., Laurberg P., Maia A.L., Rivkees S.A., Samuels M., Sosa J.A., Stan M.N. (2016). 2016 American Thyroid Association guidelines for diagnosis and management of hyperthyroidism and other causes of thyrotoxicosis. Thyroid.

[8] Moleti M., Violi M.A., Montanini D., Trombetta C., Di Bella B., Sturniolo G., Presti S., Alibrandi A., Campennì A., Baldari S. (2014). Radioiodine ablation of postsurgical thyroid remnants after treatment with recombinant human TSH (rh TSH) in patients with moderate-to-servere Graves’ orbitopathy (GO): A prospective, randomized, single-blind clinical trial. J. Clin. Endocrinol. Metab..

[9] Kim J., Choi M.S., Park J., Park H., Jang H.W., Choe J.-H., Kim J.-H., Kim J.S., Cho Y.S., Choi J.Y. (2021). Changes in thyrotropin receptor antibody levels following total thyroidectomy or radioiodine therapy in patients with refractory Graves’ disease. Thyroid.

[10] Andersen S.L., Knøsgaard L., Olsen J., Vestergaard P., Andersen S. (2019). Maternal thyroid function, use of antithyroid drugs in early pregnancy, and birth defects. J. Clin. Endocrinol. Metab..

[11] Andersen S.L., Lonn S., Vestergaard P., Torring O. (2017). Births defects after use of antithyroid drugs in early pregnancy: A Swedish nationwide study. Eur. J. Endocrinol..

[12] Abeillon-du Payrat J., Chikh K., Bossard N., Bretones P., Gaucherand P., Claris O., Charrié A., Raverot V., Orgiazzi J., Borson-Chazot F. (2014). Predictive value of maternal second-generation thyroid-binding inhibitory immunoglobulin assay for neonatal autoimmune hyperthyroidism. Eur. J. Endocrinol..

[13] Van Dijk M.M., Smits I.S., Fliers E., Bisschop P.H. (2018). Maternal thyrotropin receptor antibody concentration and the risk of fetal and neonatal thyrotoxicosis: A systematic review. Thyroid.

[14] Yoshihara A., Iwaku K., Yoshimura Noh Y., Watanabe N., Kunii Y., Ohye H., Suzuki M., Matsumoto M., Suzuki N., Tadokoro R. (2019). Incidence of neonatal hyperthyreoidism among newborns of Graves’ disease patients treated with radioiodine therapy. Thyroid.

[15] Ashkar C., Sztal-Mazer S., Topliss D.J. (2023). How to manage Graves’ disease in women of childbearing potential. Clin. Endocrinol..

[16] Van der Kaay D.C.M., Wasserman J.D., Palmert M.R. (2016). Management of neonates born to mothers with Graves’ disease. Pediatrics.

[17] Besancon A., Beltrand J., Le Gac I., Luton D., Polak M. (2014). Management of neonates born to women with Graves’ disease: A cohort study. Eur. J. Endocrinol..

[18] Chaker L., Cooper D.S., Walsh J.P., Peeters R.P. (2024). Hyperthyroidism. Lancet.

[19] Wiersinga W.M., Poppe K.G., Effraimidis G. (2023). Hyperthyroidism: Aetiology, pathogenesis, diagnosis, management, complications, and prognosis. Lancet Diabetes Endocrinol..

[20] Seo G.H., Kim T.H., Chung J.H. (2018). Antithyroid drugs and congenital malformations. A nationwide Korean cohort study. Ann. Intern. Med..

[21] Barbero P., Valdez R., Rodriguez H., Tiscornia C., Mansilla E., Allons A., Coll S., Liascovich R. (2008). Choanal atresia associated with maternal hyperthyroidism treated with methimazole: A case-control study. Am. J. Med. Genet. A.

[22] Momotani N., Ito K., Hamada N., Ban Y., Nishikawa Y., Mimura T. (1984). Maternal hyperthyroidism and congenital malformation in the offspring. Clin. Endocrinol..

[23] Agrawal M., Lewis S., Premawardhana L., Dayan C.M., Taylor P.N., Okosieme O.E. (2022). Antithyroid drug therapy in pregnancy and risk of congenital anomalies: Systemic review and meta-analysis. Clin. Endocrinol..

[24] Verburg F.A., Schmidt M., Kreissl M.C., Grünwald F., Lassmann M., Hänscheid H., Hohberg M., Luster M., Dietlein M. (2019). Guideline for I-131 whole body scintigraphy for differentiated thyroid cancer (AWMF No. 031-013). German. Nukl..

[25] Yoshihara A., Yoshimura Noh Y., Inoue K., Watanabe N., Fukushita M., Matsumoto M., Suzuki N., Suzuki A., Kinoshita A., Yoshimura R. (2023). Incidence of and risk factors for neonatal hypothyroidism among women with Graves’ disease treated with antithyroid drugs until delivery. Thyroid.

